# Protein quality of soy and the effect of processing: A quantitative review

**DOI:** 10.3389/fnut.2022.1004754

**Published:** 2022-09-27

**Authors:** Lisa A. van den Berg, Jurriaan J. Mes, Marco Mensink, Anne J. Wanders

**Affiliations:** ^1^Unilever Foods Innovation Centre, Unilever R&D, Wageningen, Netherlands; ^2^Division of Human Nutrition and Health, Wageningen University and Research, Wageningen, Netherlands; ^3^Wageningen Food and Biobased Research, Wageningen University and Research, Wageningen, Netherlands

**Keywords:** plant-based, protein quality, digestible indispensable amino acid score, protein digestibility corrected amino acid score, soy, processing

## Abstract

There is a growing demand for plant-based protein-rich products for human consumption. During the production of plant-based protein-rich products, ingredients such as soy generally undergo several processing methods. However, little is known on the effect of processing methods on protein nutritional quality. To gain a better understanding of the effect of processing on the protein quality of soy, we performed a quantitative review of *in-vivo* and *in-vitro* studies that assessed the indispensable amino acid (IAA) composition and digestibility of varying soy products, to obtain digestibility indispensable amino acids scores (DIAAS) and protein digestibility corrected amino acid scores (PDCAAS). For all soy products combined, mean DIAAS was 84.5 ± 11.4 and mean PDCAAS was 85.6 ± 18.2. Data analyses showed different protein quality scores between soy product groups. DIAAS increased from tofu, soy flakes, soy hulls, soy flour, soy protein isolate, soybean, soybean meal, soy protein concentrate to soymilk with the highest DIAAS. In addition, we observed broad variations in protein quality scores within soy product groups, indicating that differences and variations in protein quality scores may also be attributed to various forms of post-processing (such as additional heat-treatment or moisture conditions), as well as study conditions. After excluding post-processed data points, for all soy products combined, mean DIAAS was 86.0 ± 10.8 and mean PDCAAS was 92.4 ± 11.9. This study confirms that the majority of soy products have high protein quality scores and we demonstrated that processing and post-processing conditions can increase or decrease protein quality. Additional experimental studies are needed to quantify to which extent processing and post-processing impact protein quality of plant-based protein-rich products relevant for human consumption.

## Introduction

To support the transition toward more sustainable (plant-based) dietary patterns there is a growing demand for plant-based protein-rich products (1). At present, plant-based protein-rich products mainly comprise soybeans because of their low cost and high protein quality (2). A recent study by Fanelli et al. (3) for instance found that soy-based burgers had significantly higher protein quality compared to pea-based burgers. The quality of a protein source depends on the content and pattern of indispensable amino acids (IAAs) it provides, as well as the digestibility of these IAAs. To ensure protein synthesis by the body, a complete pattern of digestible IAAs is required. The amounts of IAAs that could finally be utilized by the body are dependent on the levels of amino acids present in a protein source, as well as the digestibility of these IAAs. In general, protein sources that consist of IAA patterns matching the recommended requirements for IAAs (4) and which are easily digested and absorbed by the body, have a high protein quality (5). The higher the protein quality score, the better the protein source fulfills our body's IAA requirements.

**Figure 1:**
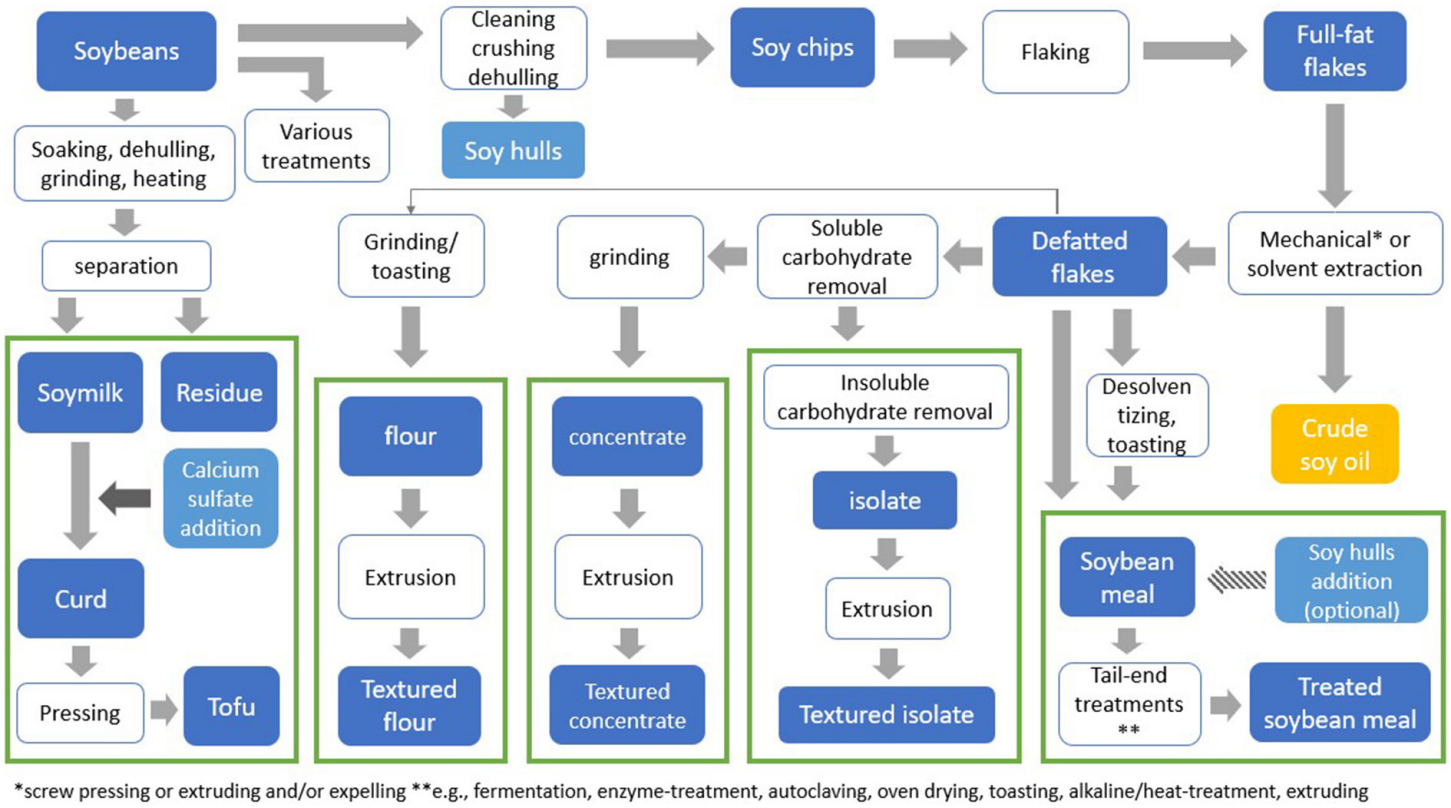
Overview of soybean processing to obtain various soy products. *Mechanical pressing or extruding and/or expelling **e.g., fermentation, enzyme-treatment, autoclaving, oven drying, toasting, alkaline-heat-treatment, extruding.

Various processing techniques are being used to obtain soy fractions needed to produce soy-based products intended for human consumption (Figure 1). Soy protein flour is obtained by cleaning, crushing, dehulling, and flaking of soybeans, followed by oil extraction. This same process, extended by the removal of soluble carbohydrates from the defatted soy flakes, is used to produce soy protein concentrate. Soy protein isolate is obtained after the additional removal of insoluble carbohydrates. Soy protein flour, concentrate and isolate are the main raw materials used in the development of plant-based meat products. To obtain textured fibrous structures that closely resemble animal meat, soy flour, concentrate and isolate undergo post-processing procedures such as extrusion (6). During extrusion, protein material goes through a series of physical and chemical changes under thermomechanical treatment. The components present in the material interact, and a meat-like structure can be formed (2).

Although soy is well known for its high protein quality, processing may influence the amino acid pattern and digestibility of soy protein, leading to different protein quality scores for different soy protein products (7). For instance, Sarwar (8) showed that protein quality differs for soy protein isolate, alkaline-heat-treated soy protein isolate, and soybean meal. In addition, Sá et al. (9) reported that different post-processing methods, such as autoclaving and fermentation, lead to differences in plant protein digestibility, suggesting variations in protein quality as well. Several potential mechanisms can explain this. For instance, heat treatment can increase protein quality by inactivating certain antinutritional factors (ANF) that limit the digestibility of protein (10). In addition, processing of plant protein sources might disrupt cell wall constituents, also leading to increased protein digestibility (11). Conversely, processing might lead to decreased protein digestibility in certain cases due to protein aggregation and reduced solubility limiting the accessibility for digestive enzymes (12, 13).

The current standard to evaluate protein quality is the digestible indispensable amino acid score (DIAAS), which was coined after addressing some limitations of the older protein digestibility-corrected amino acid score (PDCAAS). A major difference between these two protein quality scores is that the DIAAS relates the amount of ingested protein with the levels present at the end of the small intestine (“ileal digestibility”), while PDCAAS uses the levels of protein remaining in the feces (“fecal digestibility”) (4). As there is limited data on amino acid digestibility at the ileal level, the use of fecal digestibility is still a widely accepted method to evaluate protein quality and compare protein quality between sources (14).

The objective of the present review was to gain insight into the effect of processing on the protein quality of soy. Soy was selected as it currently is the most widely used and studied plant-based protein source. We performed a quantitative review of *in-vivo* and *in-vitro* studies that assessed indispensable amino acid (IAA) composition and digestibility of soy after a range of processing methods to obtain indispensable amino acid scores (DIAAS) and protein digestibility dependent amino acid scores (PDCAAS).

## Methods

### Search strategy

A literature search was conducted on PubMed, Scopus, and Ovid CAB abstracts. Titles and abstracts of articles published from January 2000 to March 2021 were searched for combinations of the following terms: protein and digestible indispensable amino acid score, DIAAS, protein digestibility corrected amino acid score, PDCAAS, or ileal digestibility. The search strategy can be found in Supplementary Figure 1.

### Study selection

The retrieved articles were screened for eligibility based on title and abstract by one of the authors (LB). After title-abstract screening, the full texts of these articles were examined. In addition, reference lists of the retrieved review articles were screened to identify additional eligible intervention studies. Intervention studies and review articles were included when they had at least one study arm with soy, and presented quantitative data on DIAAS and/or PDCAAS or on parameters that could be converted into DIAAS or PDCAAS, originating from research in humans, pigs, rats, or *in-vitro* studies. Parameters needed for DIAAS calculations were the amino acid profile of the investigational product and the standardized or true ileal digestibility (SID or TID) for each indispensable amino acid (IAA) (i.e., histidine, isoleucine, leucine, lysine, methionine + cysteine, phenylalanine + tyrosine, threonine, tryptophan, and valine). Parameters needed for PDCAAS calculations were the amino acid profile of the investigational product and the total fecal digestibility of the protein source. When protein quality scores could not be calculated due to missing values of the IAA content of the investigational products, and/or digestibility of the IAAs, the study was excluded. However, for studies that did not measure tyrosine, data was included when phenylalanine on its own was not the limiting amino acid, as phenylalanine and tyrosine together represent the aromatic amino acid (AAA) content of a product. Furthermore, studies performed in humans or animals that were pregnant, lactating, had health problems, or suffered from an abnormal digestible system were excluded.

### Data extraction

The following data was collected from each study as available: publication characteristics; study population characteristics; study methodology; type of soy product and processing details; dry matter content (%), crude protein content (%), and amino acid content (mg/g protein) of protein source; ileal digestibly (SID/TID) (%), fecal digestibility (%) and/or protein quality score (DIAAS/PDCAAS) (%); reference age pattern used for protein quality calculations.

### Calculations

For each dataset, PDCAAS or DIAAS was (re)calculated according to a standardized formula. When data on ileal digestibility and amino acid contents were not completely available, PDCAAS or DIAAS were extracted from the original publication.

#### DIAA ratio and DIAAS

For each amino acid, digestible indispensable amino acid (DIAA) ratio was calculated using the amino acid composition and standardized ileal digestibility (SID) or true ileal digestibility (TID) as follows:


(1) $$\begin{array}{l} DIAA\;ratio=\frac{mg\;of\;amino\;acid\;in\;1\;g\;test\;protein}{reference\;pattern\;score}\\ \;\times \;ileal\;digestibility\;of\;amino\;acid \end{array}$$

(2) $$\begin{array}{l} DIAAS\;\left(\%\right)=100\;\times \;lowest\;DIAA\;ratio \end{array}$$

DIAA ratios were determined according to the three reference pattern scores defined by FAO: (1) infants aged 0–0.5 years, (2) children aged 0.5–3 years, and (3) children older than 3 years, adolescents, and adults (4) (Supplementary Table 1). Comparisons of DIAA ratios and scores between soy products were based on the IAA requirements for children aged 0.5–3 years, as this is the recommended reference pattern score of human foods for regulatory purposes (4). Results for other scoring patterns can be found in Supplementary Figure 3.

#### PDCAA ratio and PDCAAS

For each amino acid, the protein digestibility corrected amino acid (PDCAA) ratio was determined by using the amino acid composition and fecal digestibility as follows:


(3) $$\begin{array}{l} PDCAA\;ratio=\frac{mg\;of\;amino\;acid\;in\;1\;g\;test\;protein}{reference\;pattern\;score}\\ \;\times \;total\;fecal\;digestibility \end{array}$$

(4) $$\begin{array}{l} PDCAAS\;\left(\%\right)=100\;\times \;lowest\;PDCAA\;ratio \end{array}$$

PDCAAS values were calculated using the reference pattern score of children aged 2–5 years (15), as this is the leading approach used in literature to obtain PDCAAS values (Supplementary Table 2).

### Data interpretation and analysis

Crude protein content, amino acid composition, amino acid ratios, and protein quality scores were presented as mean ± standard deviation (SD). Data were analyzed by SPSS version 25 for Windows (SPSS Inc., Chicago, IL, USA). Normality of the data was tested by Histograms and Q-Q plots. Homogeneity of variances was checked using Levene's statistics. One-way ANOVA was used to compare mean DIAA ratios, DIAAS, PDCAA ratios, and PDCAAS between soy product groups (i.e., soybean, soy hulls, full-fat soy flakes, soybean meal, soy protein flour, soy protein concentrate, soy protein isolate, tofu, and soymilk). A *p*-value < 0.05 was considered significant.

Besides the processing techniques used to obtain different soy products, protein quality scores of soy products after various post-processing methods were visualized to evaluate the impact of post-processing on soy protein quality. Post-processing was defined as any additional procedure that a soy product undergoes, such as additional alkaline treatment, heat-treatment, boiling, autoclaving, microbial fermentation, enzyme treatment, or extrusion.

To explore the effect of study condition (i.e., *in-vitro* versus *in-vivo* digestibility model, type of animal digestibility model) on protein quality, mean protein quality scores for soy products were obtained for different study conditions. Mean ± standard deviation (SD) of DIAAS was calculated for total soy products obtained from growing pig studies and total soy products obtained from weanling pig studies. Mean ± standard deviation (SD) of PDCAAS was calculated for total soy products obtained from adult rat studies and total soy products obtained from weanling rat studies. In addition, mean DIAAS and mean PDCAAS were calculated for non-post-processed soybean meal and non-post-processed soy protein isolate, respectively, to explore the effect of study condition using more standardized soy product groups.

**Figure 2:**
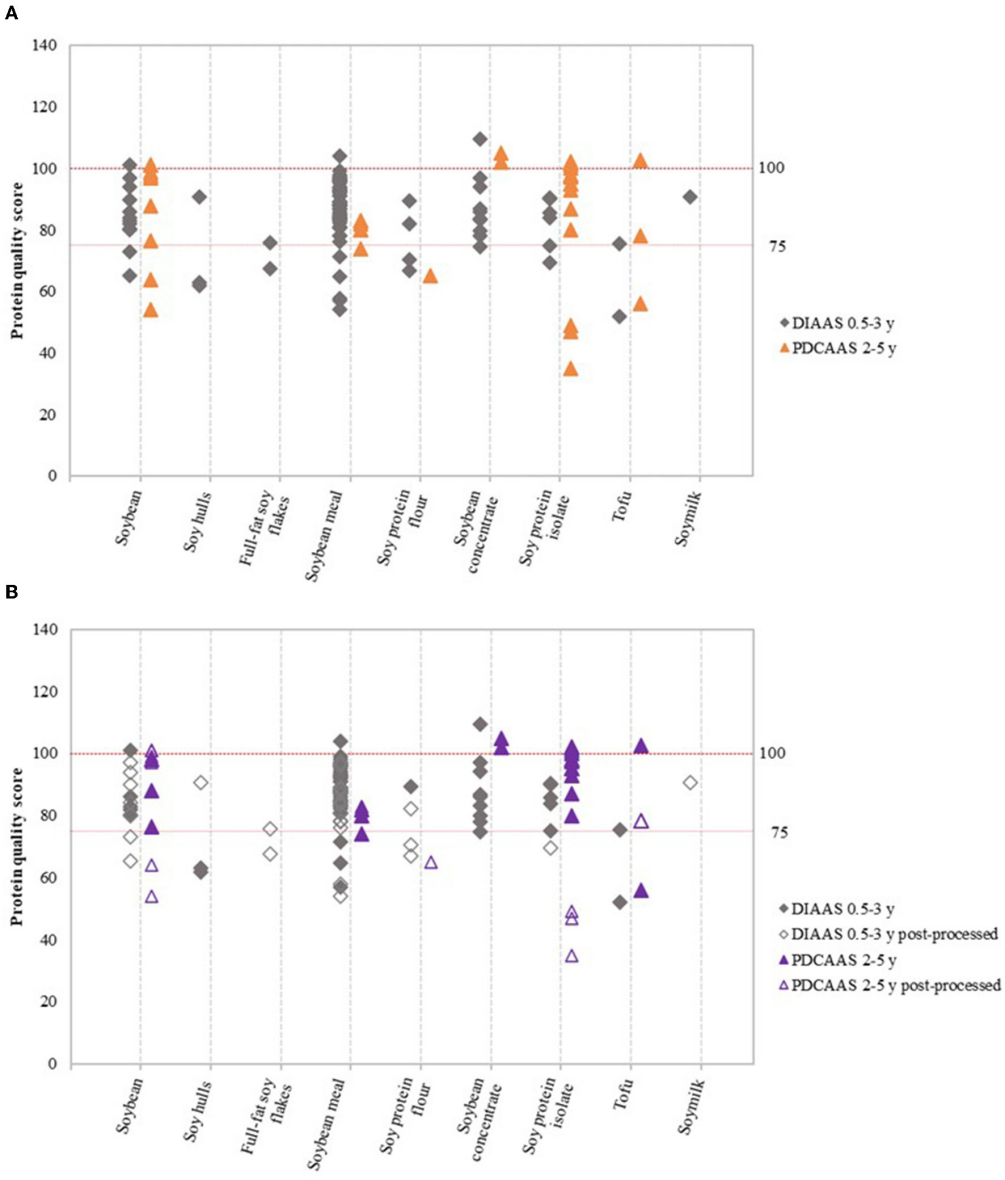
Variation in DIAAS and PDCAAS of soy product groups **(A)**, and variation in DIAAS and PDCAAS based on classification as either non-post-processed or post-processed soy product **(B)**. DIAAS calculated for reference scoring patterns of children aged 0.5–3 years [2013 FAO report (4)] and PDCAAS for reference scoring patterns of children aged 2–5 years [1985 WHO report (15)].

**Figure 3:**
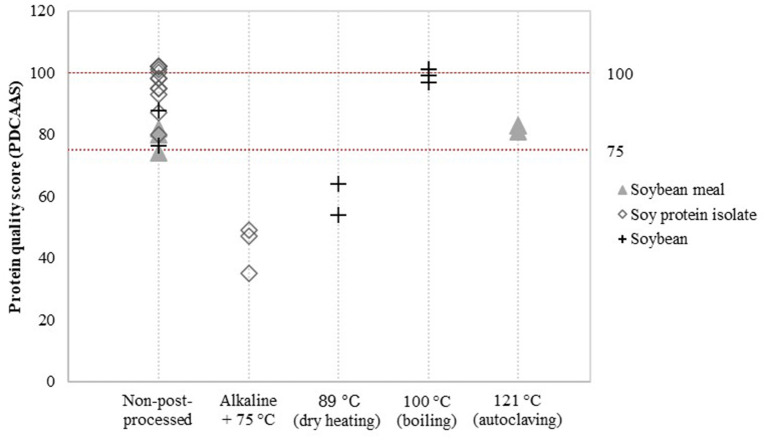
Reported PDCAAS for non-post-processed soy products and various post-processed soy products. Values derived from (8, 21–28).

**Figure 4:**
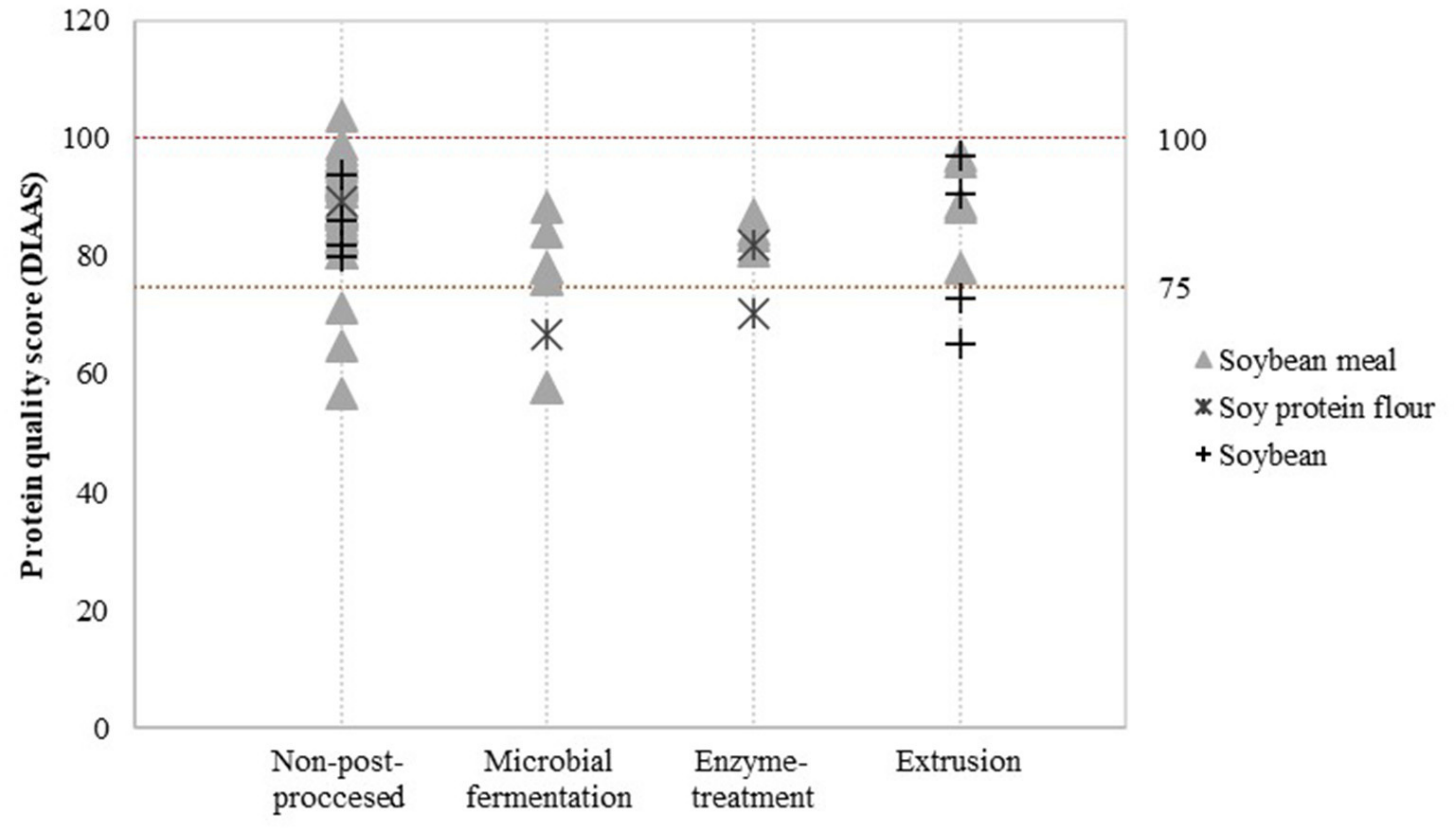
Reported DIAAS for non-post-processed soy products and various post-processed soy products. Values derived from (16–20, 29–56).

The Food and Agriculture Organization (FAO) currently recommends that high protein-quality and excellent protein-quality claims should be only made if DIAAS values based on the requirements for the age group 0.5–3 years are above 75 and 100, respectively (4). Trendlines for protein quality scores of 75 and 100 were therefore included in Figures 2–**4** to enable interpretation of the data.

## Results

### Literature search

Initially, the literature search retrieved 2,880 articles. After duplicate removal and title-abstract screening, 474 potentially eligible articles were full text reviewed. Fifty-eight additional records, including articles published before 2000, were obtained through reference screening of review articles. Finally, 45 articles met the inclusion criteria and were included in the present study. An overview of the literature screening process is shown in Supplementary Figure 2.

### Study characteristics

Thirty-three sources reported DIAAS values or data that could be recalculated into DIAAS values, of which four were major animal feed databases. Moreover, nine articles reported PDCAAS values, and three included DIAAS as well as PDCAAS values. DIAAS values were mainly obtained from studies in pigs (*n* = 34), while PDCAAS values were mainly obtained from studies in rats (*n* = 9), and *in-vitro* (*n* = 2). The literature search identified a few human digestion studies. However, none of the human studies fulfilled all inclusion criteria. In total, the 45 included sources resulted in 95 datasets for DIAAS and 38 for PDCAAS values.

The datasets represented nine soy product groups; Table 1 depicts the number of datasets by soy product group and protein quality score. In general, more DIAAS datasets were available compared to PDCAAS, except for soy protein isolate and tofu. Many DIAAS datasets were obtained for soybean meal, likely as this is a major source of animal feed. From the total number of DIAAS and PDCAAS datasets, 45 datasets were obtained for post-processed soy products. Detailed information about the included datasets can be found in Supplementary Table 4.

**Table 1:** Overview of datasets by soy product group and protein quality score.

**Soy product group**	**DIAAS**	**PDCAAS**
Soybean	12	8
Soy hulls	3	0
Full-fat soy flakes	2	0
Soybean meal	56	6
Soy protein flour	4	2
Soy protein concentrate	9	2
Soy protein isolate	6	17
Tofu	2	3
Soymilk	1	0
Total	95	38

### Variation in DIAAS and PDCAAS values

Figure 2A shows the variation in DIAAS and PDCAAS values of the nine soy product groups. Results based on the scoring patterns for infants aged 0–0.5 years, children older than 3 years, adolescents, and adults can be found in Supplementary Figure 3. Figure 2B shows the same data as Figure 2A, but DIAAS and PDCAAS data were classified as either non-post-processed or post-processed soy products. The figures show that the majority of studies on soy resulted in DIAAS and PDCAAS values above 75, which classifies as high-quality protein (4). However, substantial variations were observed in DIAAS and PDCAAS values between soy product groups as well as within soy product groups. For some product groups, values below 75 were found, especially for post-processed soy products such as post-processed soybean, soy protein flour and soy protein isolate (Figure 2B). In addition, some studies showed DIAAS values above 100 for soy products, with maximum values of 101 for soybean (16), 110 for soy protein concentrate (17), and 104 for soybean meal (18). The same products were reported to have DIAAS values as low as 65 (19), 75 (20), and 45 (20), respectively, highlighting the presence of broad variation in protein quality within product groups and between studies.

Mean DIAA ratios and DIAAS values are given in Tables 2, 3. Sulfur-containing amino acids (SAA) displayed the lowest DIAA ratio, indicating that these are in general the first limiting amino acids for soy products, followed by lysine (Table 2). ANOVA analysis indicated that DIAA ratios for histidine, isoleucine, leucine, aromatic amino acids (phenylalanine + tyrosine), threonine, tryptophan, valine (all *p* < 0.01) differed significantly among soy product groups. Considering all available data for any soy product and using the reference scoring pattern for the age of 0.5–3 years, mean DIAAS was 84.5 ± 11.4 (Table 3). When excluding post-processed soy products to reduce post-processing as a source of heterogeneity, the mean DIAAS was 86.0 ± 10.8. ANOVA indicated significantly different DIAAS values between the different soy products (*p* < 0.05).

**Table 2:** Digestible indispensable amino acid ratios^a^ (mean ± SD) for total soy and soy product groups.

	**Total soy (n = 75)**	**Soybean** **(n = 7)**	**Soy hulls (n = 3)**	**Full-fat soy flakes** **(n = 2)**	**Soybean meal** **(n = 44)**	**Soy protein flour (n = 4)**	**Soy protein concentrate** **(n = 8)**	**Soy protein isolate (n = 5)**	**Tofu (n = 1)**	**Soymilk** **(n = 1)^d^**	***P*-value ANOVA**
His	1.17 ± 0.14	1.14 ± 0.08	0.94 ± 0.16	0.97 ± 0.05	1.19 ± 0.11	1.10 ± 0.11	1.25 ± 0.17	1.22 ± 0.09	1.27	1.29	< 0.01
**Ileu**	1.23 ± 0.15	1.19 ± 0.10	0.99 ± 0.16	0.90 ± 0.01	1.23 ± 0.12	1.20 ± 0.09	1.30 ± 0.13	1.42 ± 0.09	1.38	1.37	< 0.01
**Leu**	1.00 ± 0.10	0.96 ± 0.06	0.80 ± 0.12	0.74 ± 0.01	1.01 ± 0.07	0.96 ± 0.07	1.07 ± 0.05	1.14 ± 0.08	1.10	1.04	< 0.01
**Lys**	0.94 ± 0.11	0.92 ± 0.07	0.89 ± 0.17	0.85 ± 0.00	0.94 ± 0.12	0.85 ± 0.13	0.99 ± 0.05	1.04 ± 0.06	0.93	0.91	0.095
**SAA** ^b^	0.87 ± 0.13	0.86 ± 0.13	0.83 ± 0.10	0.74 ± 0.06	0.89 ± 0.12	0.77 ± 0.09	0.90 ± 0.15	0.84 ± 0.08	0.75	1.11	0.130
**AAA** ^c^	1.42 ± 0.21	1.33 ± 0.27	1.21 ± 0.10	1.00 ± 0.03	1.43 ± 0.17	1.52^d^	1.47 ± 0.20	1.68 ± 0.11	1.48	1.41	< 0.01
**Thr**	1.04 ± 0.11	0.99 ± 0.08	0.93 ± 0.15	0.80 ± 0.02	1.05 ± 0.09	0.96 ± 0.10	1.12 ± 0.09	1.10 ± 0.11	1.08	1.10	< 0.01
**Trp**	1.35 ± 0.35	1.10 ± 0.18	0.82 ± 0.18	1.39 ± 0.05	1.35 ± 0.21	1.28 ± 0.22	1.33 ± 0.09	1.53 ± 0.16	2.80	3.07	< 0.01
**Val**	0.95 ± 0.10	0.92 ± 0.08	0.81 ± 0.11	0.74 ± 0.06	0.95 ± 0.08	0.92 ± 0.10	1.04 ±−0.05	1.04 ± 0.04	1.03	1.00	< 0.01

a Based on reference pattern scores of children aged 0.5–3 years (4).

b Sulfur-containing amino acids (methionine + cysteine).

c Aromatic amino acids (phenylalanine + tyrosine).

d No SD since only one DIAA ratio was available.

**Table 3:** DIAAS (%)^a^ (mean ± SD) for total soy and soy product groups, calculated from all available data and after excluding post-processed soy products.

		**Total soy**	**Soybean**	**Soy hulls**	**Full-fat soy flakes**	**Soybean meal**	**Soy flour**	**Soy protein concentrate**	**Soy protein isolate**	**Tofu**	**Soymilk**	***P*-value** **ANOVA**
**All data**	**n**	**95**	12	3	2	56	4	9	6	2	1	
	**DIAAS** ^**b**^	84.5 ± 11.4	84.6 ± 9.6	71.9 ± 13.3	71.7 ± 4.2	86.5 ± 10.8	77.2 ± 9.0	87.7 ± 10.3	82.4 ± 7.7	63.7 ± 11.7	90.7	0.012
**Non-post-processed data** ^**c**^	**n**	67	5	2	2	41	1	9	5	2		
	**DIAAS**	86.0 ± 10.8	86.4 ± 7.5	62.4 ± 0.6	71.7 ± 4.2	88.6 ± 8.8	89.4	87.7 ± 10.3	85.0 ± 5.6	63.7 ± 11.7	na^d^	< 0.01

a Based on reference pattern scores of children aged 0.5–3 years (4).

b DIAAS might differ from the lowest DIAA ratio for the same product group in Table 2 as all values are averages based on the **total number of datasets (n)** included for the specific outcome.

c Data from post-processed soy products excluded.

d na, not available.

Mean PDCAA ratios and PDCAAS values are given in Tables 4, 5. For PDCAAS, lysine was observed to be the first limiting amino acid for total soy, followed by SAA (Table 4). Mean PDCAAS values obtained for total soy products and non-post-processed total soy products were 85.6 ± 18.2 and 92.4 ± 11.9, respectively (Table 5). Differences between soy product groups were significant when assessing non-post-processed data (*p* = 0.028) but not when assessing all available data (*p* = 0.744). In general, large variations within soy product groups were observed for all outcomes, indicating that not only the difference in type of soy product impacts protein quality scores but also that factors such as post-processing have an effect.

**Table 4:** Protein digestibility corrected amino acid ratios^a^ (mean ± SD) for total soy and soy products.

	**Total soy (n = 20)**	**Soybean (n = 8)**	**Soybean meal**	**Soy protein flour (n = 1)**	**Soy protein concentrate (n = 2)**	**Soy protein isolate** **(n = 11)**	**Tofu**	***P*-value ANOVA**
**His**	1.30 ± 0.22	1.20 ± 0.04	na^b^	1.30	1.27 ± 0.08	1.36 ± 0.27	na	0.672
**Ileu**	1.55 ± 0.14	1.42 ± 0.11	na	1.44	1.55 ± 0.06	1.61 ± 0.12	na	0.013
**Leu**	1.13 ± 0.09	1.03 ± 0.06	na	1.04	1.11 ± 0.02	1.18 ± 0.05	na	< 0.01
**Lys**	1.01 ± 0.06	0.95 ± 0.06	na	0.98	1.04 ± 0.02	1.04 ± 0.04	na	0.376
**SAA** ^c^	1.03 ± 0.12	1.15 ± 0.11	na	1.00	1.08 ± 0.03	0.96 ± 0.07	na	0.822
**AAA** ^d^	1.31 ± 0.12	1.17 ± 0.10	na	1.21	1.33 ± 0.03	1.38 ± 0.06	na	0.004
**Thr**	1.04 ± 0.09	0.99 ± 0.12	na	1.01	1.03 ± 0.02	1.07 ± 0.06	na	0.642
**Trp**	1.24 ± 0.29	1.12 ± 0.12	na	1.24	1.09 ± 0.08	1.33 ± 0.34	na	0.534
**Val**	1.28 ± 0.11	1.16 ± 0.06	na	1.24	1.31 ± 0.02	1.35 ± 0.08	na	< 0.01

a Based on reference pattern score of children aged 2–5 years (15).

b na, not available.

c Sulfur-containing amino acids (methionine + cysteine).

d Aromatic amino acids (phenylalanine + tyrosine).

**Table 5:** PDCAAS (%)^a^ (mean ± SD) for total soy and soy product groups calculated from all available data and after excluding post-processed soy products (untruncated).

		**Total soy**	**Soybean**	**Soybean meal**	**Soy flour**	**Soy protein concentrate**	**Soy protein isolate**	**Tofu**	***P*-value ANOVA**
**All data**	**n**	38	8	6	2	2	17	3	
	**PDCAAS** ^**b**^	85.6 ± 18.2	85.0 ± 17.2	81.0 ± 3.4	81.3 ± 16.6	103.4 ± 1.9	87.2 ± 20.9	78.9 ± 19.0	0.744
**Non-post- processed data** ^**c**^	**n**	24	2	3	1	2	14	2	
	**PDCAAS**	92.4 ± 11.9	82.2 ± 5.5	79.7 ± 3.9	97.9	103.4 ± 1.9	96.5 ± 6.1	79.3 ± 23.3	0.028

a Based on reference pattern score of children aged 2–5 years (15).

b PDCAAS might differ from the lowest PDCAA ratio for the same product group in Table 4 as all values are averages based on the **total amount of datasets (n)** included for the specific outcome.

c Data from post-processed soy products excluded.

### Post-processing

Detailed assessment of the impact of post-processing on protein quality was possible for a limited number of post-processing treatments and soy product groups. Data was available to evaluate the effect of additional heat treatment on PDCAAS values (Figure 3) and the effect of fermentation, enzyme treatment and extrusion on DIAAS values (Figure 4).

Alkaline-heat-treated soy protein isolate (75 °C) showed relatively low PDCAAS values compared to non-post-processed soy protein isolate (Figure 3). PDCAAS values for alkaline-heat-treated soy protein isolate were obtained from two studies (8, 21), resulting in a mean value for alkaline-heat-treated soy protein isolate of 43.7 ± 6.2, whereas the mean value of non-post-processed soy protein isolate was 96.5 ± 6.1 (Table 5). Furthermore, Pires et al. (22) treated soybean by dry heating at temperatures of 89 °C. They reported PDCAAS values of 54 and 64 for two different soybean cultivars after dry heating, which are lower compared to the mean value of 82.2 for untreated soybean obtained by Stone et al. (23). In contrast, boiling soybeans (100 °C) was found to increase PDCAAS values to scores of approximately 100 (24). Additional autoclaving of soybean meal to 121 °C did not have a profound effect on the PDCAAS value (8, 21).

Figure 4 indicates that there may be minor differences in DIAAS values after applying post-processing treatments. Compared to non-post-processed soy protein flour, microbial fermentation and enzyme treatment seems to slightly decrease the DIAAS of soy protein flour. Nevertheless, this effect was not observed for soybean meal. DIAAS values for extruded soybeans and soy products vary between studies, ranging from a DIAAS for extruded soybean of 65 calculated from a study by Urbaityte et al. (20) to a score of 97 obtained by Cervantes-Pahm and Stein (29).

### Study conditions

We performed explorative analyses to investigate the effect of study conditions on protein quality, though it should be noted that sample numbers were small (Supplementary Tables 5, 6). DIAAS values for soybean meal without reported post-processing treatments were observed to be similar between studies in growing pigs and weanling pigs. The overall mean PDCAAS obtained from adult rat studies for total non-post-processed soy was lower than the mean PDCAAS obtained from weanling rat studies (85.7 ± 17.6 vs. 95.8 ± 8.6, respectively). When limiting the soy products to non-post-processed soy protein isolate, the mean PDCAAS was also observed to be similar between adult rat and weanling rat studies.

## Discussion

In this quantitative review of *in-vivo* and *in-vitro* studies on protein quality (i.e., DIAAS and/or PDCAAS) of soy, we observed that the majority of soy products have a high protein quality, but the scores differed between soy product groups as well as within soy product groups (i.e., soybean, soy hulls, full-fat soy flakes, soybean meal, soy protein flour, soy protein concentrate, soy protein isolate, tofu, and soymilk). We showed that the differences between and variations within product groups may be attributed to various forms of processing, post-processing, as well as study conditions.

### Processing into soy products

To obtain different soy products, sets of constituents are being removed during the production process, which may impact protein quality (14). For instance, soy flour is produced by grinding defatted soy flakes. Soy protein concentrate is obtained similarly but requires additional removal of soluble carbohydrates from defatted soy flakes. Consequently, soy protein concentrate contains less starch, fiber, antinutritional factors (ANFs) such as trypsin inhibitors, and a higher crude protein level (Supplementary Table 4). Several of these components are known to obstruct optimal protein digestion, leading to lower protein quality scores of protein flour compared to protein concentrate (14, 57). This might explain the generally higher protein quality scores for soy protein concentrate (mean DIAAS of 87.7 ± 10.3 and mean PDCAAS of 103.4 ± 1.9) compared to soy protein flour (mean DIAAS of 77.2 ± 9.0 and mean PDCAAS of 81.3 ± 16.6) in the present quantitative review. Isolates have an even higher protein purity compared to concentrates, as they contain fewer carbohydrates, lactose, and fat. However, soy protein isolate did not show substantially higher protein quality scores compared to soy protein concentrate. Literature suggests that the extensive processing needed to obtain protein isolate might induce molecular alterations, making the protein more resistant to digestive enzymes and decreasing its protein nutritive value (14, 58).

Tofu is obtained after pressing of curd, which is formed by the coagulation of soymilk after calcium sulfate addition. It has been suggested that this coagulation process leads to an increase in endogenous ileal amino acid loss, and consequently leading to lower true ileal digestibility values, and higher protein quality scores of soymilk compared to tofu (30).

### Post-processing

Our findings on post-processing indicate that the temperature used for post-heating soybean products may impact protein quality. Heating soybean at temperatures of 100 °C by boiling was observed to potentially increase protein quality, while dry heating at 89 °C seemed to lower the protein quality of soybean. The higher temperatures might lead to better disruption of the rigid structure of plant protein sources, increasing the amount of smaller and better digestible particles. Moreover, higher temperatures are found to inactivate higher amounts of ANFs, resulting in improved digestibility and quality (59). However, Rehman and Shah (57) found a decrease in protein digestibility of legumes when increasing cooking time (from 10 to 90 min) and temperature (from 121 to 128 °C), which was probably due to a reduction in available lysine. Lysine may undergo Maillard reactions with reducing sugars during heat processing. This may cause a decrease in lysine contents, and hence protein quality (60). Correspondingly, González-Vega et al. (61) showed that autoclaving at 125 °C induces Maillard reactions in soybean meal. Another study, however, reported that Maillard reactions especially occur during dry heating processing, such as roasting and micronization (10, 62). Thus, not only temperature but also the heating conditions might affect protein quality. The presence of Maillard reactions due to different processing methods and the impact on protein quality need attention in future research.

Furthermore, soy protein isolate that was alkaline-heat-treated showed lower protein quality scores compared to non-post-processed soy protein isolate. Treating proteins with alkaline is known to result in the formation of lysinoalanine (LAL), which causes destruction of the indispensable amino acids lysine, cysteine, and threonine, and subsequently decreases protein digestibility (63).

Enzyme treatment and microbial fermentation did not seem to increase the protein quality of soy protein flour in the current review. However, an earlier study found that fermentation increased crude protein amounts, eliminated trypsin inhibitors, and reduced peptide size in soybeans and soybean meal (64), thus could have a beneficial impact on protein digestibility (7). Nevertheless, our findings indicate that the effect of fermentation on protein quality is not fully clear and might depend on the investigational soy product used, as well as on other factors, such as the type of microorganisms added for fermentation (20).

Full-fat soy flakes displayed relatively low protein quality scores (mean DIAAS of 71.7 ± 4.2). However, data for full-fat soy flakes were obtained from only a single study in which the full-fat soy flakes were micronized, meaning that they were additionally cooked with infrared radiant energy at approximately 105 °C for 50 s (31). In a study by Khattab and Arntfield (10), additional micronization resulted in reduced *in-vitro* protein digestibility of cowpea, pea, and kidney bean. Thus, non-post-processed full-fat soy flakes may have higher protein quality scores.

Our data showed that the effect of extrusion on protein quality differed among studies. As extrusion is a process that includes heating, it might increase protein quality by changing the protein structure and inactivation of ANFs, and decrease protein quality by inducing Maillard reactions. Furthermore, the effect of extrusion is suggested to vary per amino acid. For instance, extrusion was found to enhance the amount and digestibility of SAA but lower the amount of digestible AAA (65). Additionally, lysine loss was observed to take place during the extrusion process (66). The impact of extrusion on protein quality might therefore depend on the type and amount of limiting indispensable amino acid present in the protein source before extrusion, leading to different effect sizes of extrusion on different plant-protein sources. Moreover, moisture content might influence the effect of extrusion on protein quality. It has been observed that high moisture extrusion reduced more ANFs and led to higher protein quality compared to dry heating (67, 68). In addition, Osen et al. (69) showed that high moisture extrusion of pea proteins led to fewer Maillard reactions than low moisture extrusion. Although extrusion is the most used method of texturization, only one study (22) reported an (*in-vitro)* PDCAAS value for textured soy protein. Their observed PDCAAS value of 65 for textured soy protein was higher compared to heat-treated soybean in the same study. Unfortunately, this article did not mention details on the applied extrusion method.

### Study conditions

Besides differences in soy processing and post-processing, other factors might impact the observed protein quality scores. First, DIAAS and PDCAAS values are obtained *via* different approaches. DIAAS is based on the true ileal protein digestibility, while the PDCAAS is calculated using the true fecal digestibility. Contrary to the true ileal digestibility, the true fecal digestibility does not take into account the metabolism of unabsorbed amino acids by microorganisms present in the colon. As a result, fecal protein digestibility may overestimate true ileal protein digestibility (4) and untruncated PDCAAS values might be higher than untruncated DIAAS values (70).

In addition, the variation in protein quality scores that we observed for similar soy products, indicates that study conditions play a role. Currently, several different *in-vitro* digestion methods are used to obtain protein digestion values of food samples. However, many studies obtained from our literature search reporting *in-vitro* protein digestibility scores of soy products failed to provide digestibility scores of the complete amino acid profile. Consequently, no protein quality scores could be calculated. Nevertheless, previous studies comparing *in-vivo* with *in-vitro* methods have reported good correlations between *in-vivo* and *in-vitro* digestibility (7, 71–73), ranging from 0.7507 to 0.9649, suggesting that *in-vitro* methods for determining protein quality may be promising. However, Hughes et al. (25) compared PDCAAS values for soy using protein and amino acid profile determination by two different laboratories using different methods. Discrepancies in obtained protein and amino acid values were found, leading to variations in PDCAAS. This highlights the importance of the development of a more harmonized and standardized *in-vitro* model with clearly elaborated standard operating procedures. Previous efforts have been made to develop and describe such harmonized protocols for *in-vitro* protein digestibility, such as the INFOGEST protocol (74). However, this INFOGEST protocol still lacks a consensus to apply it for *in-vitro* DIAAS analysis for which several methods have been used and suggested (75). Further research is needed to optimize and validate these protocols for their utility in predicting protein quality scores of different protein sources. Furthermore, differences in the animal model used might lead to variations in protein quality outcomes. Gilani and Sepehr (21) emphasized the importance of standardization of the animal model. They observed considerably lower fecal protein digestibility in 5-week-old rats compared to weanling rats, leading to lower protein quality scores.

## Future research

In this study, we obtained as much data as possible for a single protein source, i.e., soy, enabling comparisons on the impact of different processing and post-processing methods as well as study conditions on protein quality. Unfortunately, many studies did not report details of processing and post-processing methods and could therefore not be utilized to the fullest. To obtain a better understanding of factors influencing variation in protein quality scores, future studies should aim to report factors such as study conditions, type of protein source, and exact processing and post-processing methods used.

This review retrieved a very limited number of studies investigating the protein quality of plant-based protein products in forms as consumed by humans such as tofu, soymilk or extruded soy concentrates. Such data are needed to be able to understand the protein quality of consumer-ready products, and to improve it when needed.

Finally, more research is needed to build general knowledge on the influence of ANF's, macro-and micronutrients, and other matrix components as well as the interplay with processing methods on protein quality of diverse plant-based protein sources, like those derived from legumes, seeds, nuts, or novel sources such as micro-organisms.

## Conclusions

In this quantitative review, we confirmed that the majority of soy products have high protein quality scores, and we demonstrated that processing and post-processing conditions can increase or decrease protein quality. The exact effects of processing and post-processing could not be quantified but depend, among others, on the specific soy product or protein fraction, temperature, and moisture conditions. Moreover, other sources of variation, including study conditions impact protein quality scores. Additional experimental studies are needed to investigate to which extent processing impacts protein quality of plant protein sources relevant for human consumption, and how to optimize protein quality of plant-based products.

## Author contributions

LvB performed the literature search, extracted the data, and conceptualized the manuscript. AW provided support in these processes. AW, JM, and MM contributed to reviewing and editing the manuscript. All authors contributed to the article and approved the submitted version.

## Conflict of interest

Author AW is an employee of Unilever. Author LvB was an internship student at Unilever for her Master in Nutrition and Health, Wageningen University, at the time of conducting the study. Unilever markets food products made from plant-based proteins. The remaining authors declare that the research was conducted in the absence of any commercial or financial relationships that could be construed as a potential conflict of interest.

## Publisher's note

All claims expressed in this article are solely those of the authors and do not necessarily represent those of their affiliated organizations, or those of the publisher, the editors and the reviewers. Any product that may be evaluated in this article, or claim that may be made by its manufacturer, is not guaranteed or endorsed by the publisher.
