# Supplementary material for: Protein quality of soy and the effect of processing: A quantitative review
Source: Front Nutr. 2022 Sep 27;9:1004754. doi: 10.3389/fnut.2022.1004754 (PMC9552267; doi:10.3389/fnut.2022.1004754)
Supplement: Supplementary file 1 [file Data_Sheet_1.pdf]

Supplementary Material

INDEX

Supplementary Figure 1 2
Supplementary Table 1 2
Supplementary Table 2 3
Supplementary Figure 2 3
Supplementary Figure 3 3
Supplementary Table 3 4
Supplementary Table 4 4
Supplementary Table 5 10
Supplementary Table 6 10

(Exposure)
(protein*) [tiab]

(Outcome)
AND
(Digestible indispensable amino acid score) [tiab] OR
(DIAAS) [tiab] OR
(Protein digestibility corrected amino acid score) [tiab] OR
(PDCAAS) [tiab] OR
(Ileal AND digestibility) [tiab]

(Limits)
NOT (broiler*) [tiab]

Supplementary Figure 2: Search strategy.

Supplementary Table 1: Amino acid scoring patterns for DIAAS calculations from the 2013 FAO report [13].

**Amino acid (mg/g crude protein)**	**Infant (0-0.5 years)**	**Child (0.5-3 years)**	**Child (>3 years), adolescent, adult**
**Histidine**	21	20	16
**Isoleucine**	55	32	30
**Leucine**	96	66	61
**Lysine**	69	57	48
**SAA**	33	27	23
**AAA**	94	52	41
**Threonine**	44	31	25
**Tryptophan**	17	8.5	6.6
**Valine**	55	43	35

Supplementary Table 2: Amino acid scoring patterns for PDCAAS calculations from the 1985 WHO report [19].

**Amino acid (mg/g crude protein)**	**Pre-school child (2-5 years)**	**School child (10-12 years)**	**Adult**
**Histidine**	19	19	16
**Leucine**	28	28	13
**Isoleucine**	66	44	19
**Lysine**	58	44	16
**SAA**	25	22	17
**AAA**	63	22	19
**Threonine**	34	28	9
**Tryptophan**	11	9	5
**Valine**	35	25	13

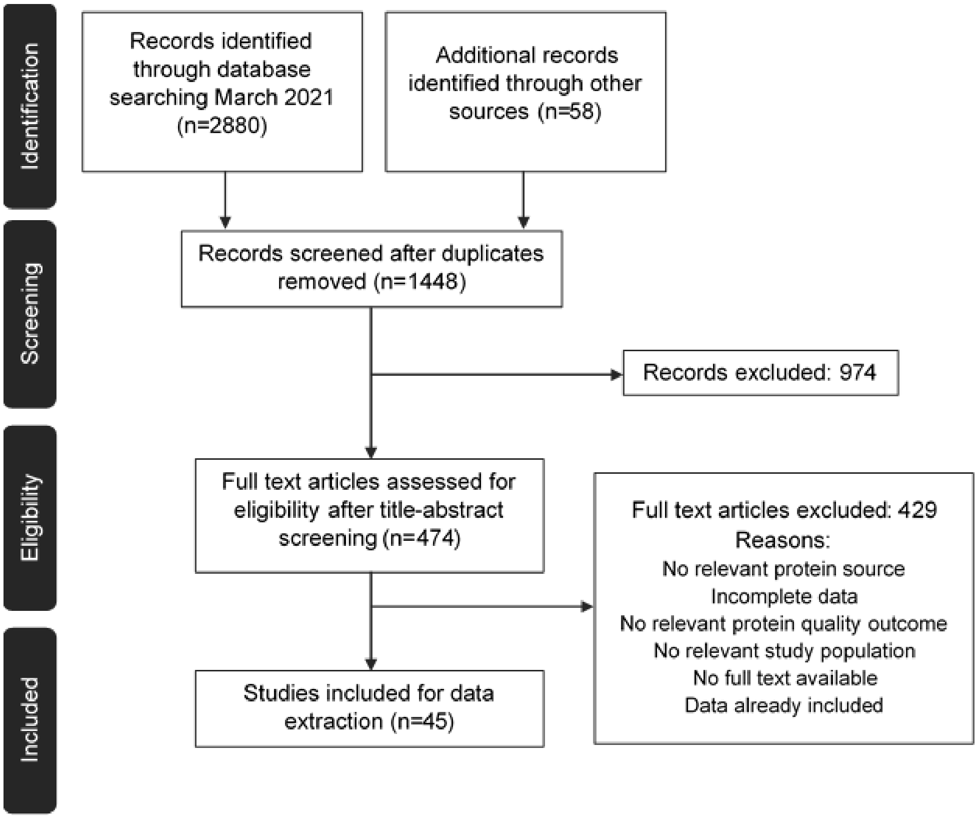

Supplementary Figure 2: Flowchart of literature screening.

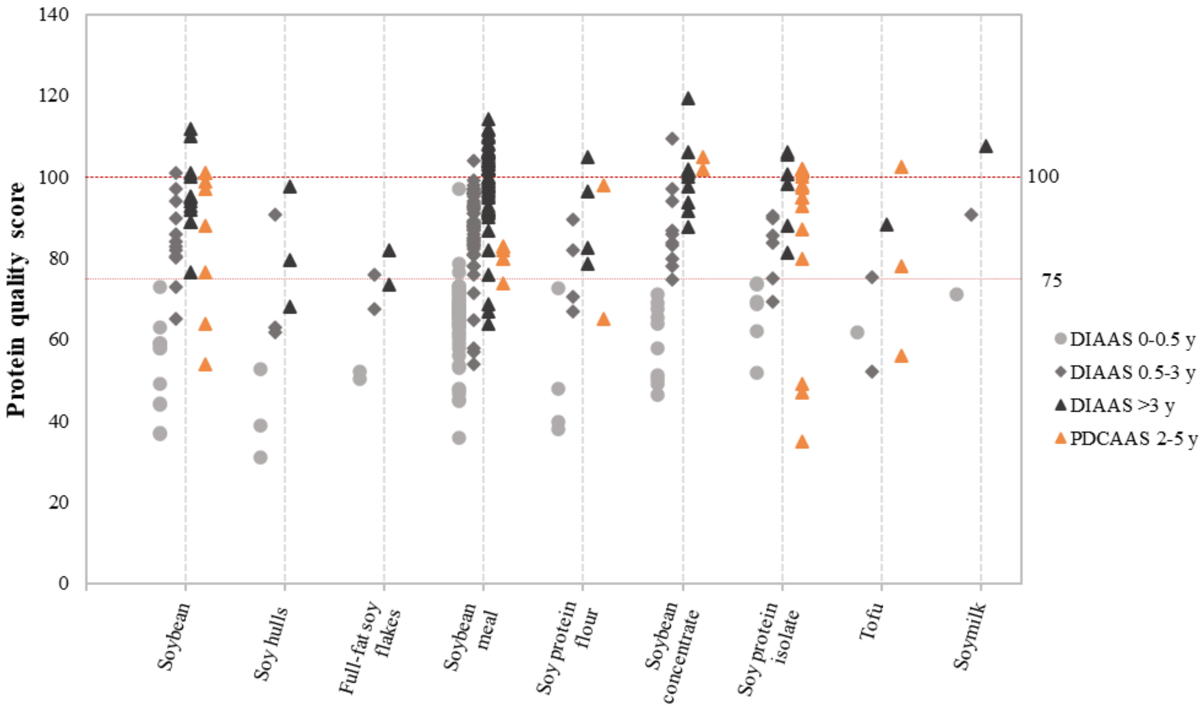

Supplementary Figure 3: Variation in DIAAS and PDCAAS of different soy products obtained from in-vivo and in-vitro studies. DIAAS calculated for three reference scoring patterns (2013 FAO report [13]) and PDCAAS for reference scoring pattern of children aged 2-5 year (1985 WHO report [19]).

Supplementary Table 3: Crude protein (mean ± SD) of soy product groups.

	Soybean (n=17)	Soy hulls (n=3)	Full-fat soy flakes (n=2)	SBM (n=54)	Soy flour (n=5)	SPC (n=12)	SPI (n=12)	Tofu (n=3)	Soymilk (n=1)	Total soy (n=109)
**Crude protein (% wet matter)**	38.45 ± 3.24	10.31 ± 0.99	40.16 ± 0.03	48.45 ± 4.19	54.50 ± 2.18	66.82 ± 4.50	84.38 ± 5.72	14.47 ± 5.84	3.82	50.60 ± 17.39

Supplementary Table 4: Reported digestible indispensable amino acids scores (DIAAS) and protein digestibility corrected amino acid scores (PDCAAS) (untruncated) for different soy products. DIAAS based on reference pattern score of children aged 0.5-3 years (2013 FAO report [1]). PDCAAS based on reference pattern score of children aged 2-5 years (1985 WHO report [2]).

Product	Additional information	Post-processing of product	Type of study	Crude protein (% wet matter)	DIAAS	PDCAAS	Limiting AA	References
**Soybean**			*In-vitro*	37.6		77	Thr	[3]
			In-vitro	37.2		88	Lys	[3]
			Growing pigs	38.6	80		AAA	[4]
	Conventional soybean		Growing pigs	37.6	82		n/a	[5]
	High-protein soybean		Growing pigs	42.8	86		n/a	[5]
	Low-oligosaccharide soybean		Growing pigs	39.3	101		n/a	[5]
		Heat-treated	Pigs	36.3	84		SAA	[6]
		Wet heated (100 °C, 16 min) + expanded (125°C, 15 sec) + autoclaved (110 °C, 60 min)	Growing pigs	37.3	79		SAA	[7]
		Treated (*unknown*)	Pigs	35.8	90		SAA	[8]
	Soybean variety SB 19	Boiled (100°C)	Weanling rats	35.2		101	Lys	[9]
	Soybean variety SB 25	Boiled (100°C)	Weanling rats	35.5		99	Lys	[9]
	Soybean variety SB 30	Boiled (100°C)	Weanling rats	34.6		97	Lys	[9]
	Soybean variety SB 132	Boiled (100°C)	Weanling rats	36.7		101	Lys	[9]
	Conventional soybean	Extruded (150 °C)	Growing pigs	35.8	97		Trp	[10]
	High-protein soybean	Extruded (150 °C)	Growing pigs	47.6	73		Trp	[10]
		Extruded	Piglets	39.2	65		SAA	[11]
		Extruded	Growing pigs	34.8	94		n/a	[12]

Product	Additional information	Post-processing of product	Type of study	Crude protein (% wet matter)	DIAAS	PDCAAS	Limiting AA	References
	Conventional soybean	Dry heated (89 °C ,5 min)	Rats	41.9		54	SAA	[13]
	Kunitz trypsin inhibitor and lipoxygenase free soybean	Dry heated (89 °C, 5 min)	Rats	40.0		64	SAA	[13]
		Toasted	Growing pigs	35.2	83		n/a	[12]
**Soy hulls**			Pigs	11.5	63		Leu	[6]
			Growing pigs	9.8	62		Trp	[14]
		Extruded (80 °C)	Growing pigs	9.7	91		Leu	[14]
**Full-fat soy flakes**	Low-oligosaccharide soybean flakes	Cooked at infrared radiant energy (105 °C, 50 sec)	Grower pigs	40.2	76		Leu	[15]
	Regular-oligosaccharide soybean flakes	Cooked at infrared radiant energy (105 °C, 50 sec)	Grower pigs	40.1	68		SAA	[15]
**Soybean meal**			Growing pigs	48.3	94		SAA	[16]
			Grower pigs	46.1	84		SAA	[17]
	Conventional soybean meal		Growing pigs	48.4	96		SAA	[18]
	High-protein soybean meal		Growing pigs	55.7	97		Leu	[18]
	Conventional soybean meal		Weanling pigs	47.8	87		SAA	[19]
			Finishing pigs	49.8	83		SAA	[20]
	Soybean meal from Korea 1		Growing pigs	47.1	57		SAA	[21]
	Soybean meal from Korea 2		Growing pigs	47.4	81		Trp	[21]
	Soybean meal from India		Growing pigs	39.6	65		SAA	[21]
			Growing pigs	45.2	85		SAA	[22]
			Growing pigs	53.1	86		SAA	[23]
	Soybean meal, expelled		Pigs	43.8	93		SAA	[6]
	Soybean meal (crude fiber <45 – crude protein >490 g/kg), solvent extracted		Pigs	48.5	96		Lys	[6]
			Weanling pigs	44.5	92		SAA	[24]
			Weanling pigs	45.0	88		SAA	[25]
		Microbially Fermented	Weanling pigs	53.9	84		Lys	[26]
	Conventional soybean meal		Weanling pigs	50.2	82		SAA	[26]
			Weanling pigs	47.7	88		SAA	[27]

Product	Additional information	Post-processing of product	Type of study	Crude protein (% wet matter)	DIAAS	PDCAAS	Limiting AA	References
			Growing pigs	42.9	93		Val	[10]
			Growing pigs	47.1	84		SAA	[28]
			Growing pigs	49.5	83		SAA	[29]
			Growing pigs	43.6	94		SAA	[14]
			Growing pigs	n/a	93		Val	[30]
			Weanling pigs	45.1	85		Val	[31]
			Growing pigs	45.6	71		Trp	[32]
			Grower pigs	47.2	96		SAA	[15]
	Soybean meal dehulled, expelled		Growing pigs	45.1	91		n/a	[5]
	Soybean meal dehulled solvent extracted		Growing pigs	47.7	92		n/a	[5]
	Soybean meal expelled		Growing pigs	44.6	91		n/a	[5]
	High-protein soybean meal dehulled, solvent extracted		Growing pigs	51.2	89		n/a	[5]
	High-protein soybean meal expelled		Growing pigs	56.0	89		n/a	[5]
	Soybean meal solvent extracted		Growing pigs	43.9	86		n/a	[5]
	Low oligosaccharide soybean meal expelled		Growing pigs	49.3	97		n/a	[5]
			Growing pigs	44.8	99		Val	[33]
	GMO-free soybean meal		Growing pigs	47.0	81		SAA	[34]
			Growing pigs	43.3	104		SAA	[35]
	Soybean meal 46		Growing pigs	43.4	96		n/a	[12]
	Soybean meal 48		Growing pigs	45.3	96		n/a	[12]
	Soybean meal 50		Growing pigs	47.2	98		n/a	[12]
			Early weaned piglets	46.2	88		Leu	[36]
		Microbially fermented	Early weaned piglets	56.2	88		Lys	[36]
		Microbially Fermented	Growing pigs	42.5	58		Lys	[37]

Product	Additional information	Post-processing of product	Type of study	Crude protein (% wet matter)	DIAAS	PDCAAS	Limiting AA	References
		Microbially Fermented	Growing pigs	54.1	76		n/a	[5]
		Microbially Fermented	Weanling pigs	53.7	78		Lys	[31]
		Enzyme-treated	Growing pigs	55.6	81		n/a	[5]
	Enzyme-treated soybean meal 1	Enzyme-treated	Weanling pigs	56.8	85		SAA	[19]
	Enzyme-treated soybean meal 2	Enzyme-treated	Weanling pigs	52.1	84		SAA	[19]
		Enzyme-treated	Weanling pigs	54.4	87		Lys	[31]
			Weanling rats	n/a		82	Trp	[38]
		Autoclaved (121 °C, 20 min)	Weanling rats	n/a		85	Lys	[38]
			Young rats	n/a		82	Trp	[39]
			Adult rats	n/a		74	Trp	[39]
		Autoclaved (121 °C, 20 min)	Young rats	n/a		83	Lys	[39]
		Autoclaved (121 °C, 20 min)	Adult rats	n/a		80	Lys	[39]
		Secondary toasted (95-100 °C) in presence of lignosulfonate	Growing pigs		45		Lys	[23]
	Soybean meal <5% crude fiber		Pigs	47.1	95		SAA	[8]
	Soybean meal >5% crude fiber		Pigs	44.6	88		SAA	[8]
	Soybean meal	Extruded	Pigs	47.4	96		Lys	[8]
	Conventional soybean meal	Extruded (145 °C)	Growing pigs	47.1	89		SAA	[18]
	High-protein soybean meal	Extruded (145 °C)	Growing pigs	56.0	88		SAA	[18]
	Low-oligosaccharide soybean meal	Extruded (145 °C)	Growing pigs	49.3	97		SAA	[18]
**Soy protein flour**			Growing pigs		89	98	Lys, SAA, respectively	[40]
	Soy protein B	Microbially fermented	Piglets	55.7	67		Lys	[11]
	Soy protein A1	Enzymatically fermented	Piglets	53.3	70		SAA	[11]
	Soy protein A3	Enzymatically fermented	Piglets	57.7	82		SAA	[11]
		Textured	Adult rats			65	SAA	[13]
**Soy protein concentrate**			Weanling pigs	62.1	80		SAA	[19]
			Pigs	65.4	110		Lys	[8]

Product	Additional information	Post-processing of product	Type of study	Crude protein (% wet matter)	DIAAS	PDCAAS	Limiting AA	References
	Soy protein concentrate + phytase		Weanling pigs	61.2	87		SAA	[27]
			Growing pigs	64.2	97		SAA	[10]
			Growing pigs	65.2	86		n/a	[5]
	Soy protein concentrate Lab A		Weanling rats	74.6		106	Thr	[41]
	Soy protein concentrate Lab B		Weanling rats	74.7		101	Trp	[41]
	Soy protein concentrate A, coarse		Piglets	65.4	83		SAA	[11]
	Soy protein concentrate B, coarse		Piglets	66.5	78		SAA	[11]
	Soy protein concentrate B, fine		Piglets	71.9	75		SAA	[11]
			Early weaned pigs	66.3	94		Lys	[36]
**Soy protein isolate**	Soy protein isolate A		Growing rats	n/a	90	102	SAA	[42]
	Soy protein isolate B		Growing rats	n/a	90	97	SAA	[42]
			Weanling pigs	77.9	86		SAA	[31]
			Growing pigs	84.8	75		n/a	[5]
			Weanling rats	n/a		80	SAA	[43]
			Rats	84.2		87	SAA	[44]
	Soy protein isolate 1 (Lab A)		Weanling rats	86.5		102	SAA	[41]
	Soy protein isolate 1 (Lab B)		Weanling rats	87.5		95	SAA	[41]
	Soy protein isolate 2 (Lab A)		Weanling rats	85.9		102	SAA	[41]
	Soy protein isolate 2 (Lab B)		Weanling rats	87.0		95	SAA	[41]
	Soy protein isolate 3 (Lab A)		Weanling rats	85.7		102	SAA	[41]
	Soy protein isolate 3 (Lab B)		Weanling rats	86.7		98	SAA	[41]
			Growing pigs	92.7	84	93	SAA	[40]
		Hydrolyzed	Piglets	84.1	69		SAA	[11]
			Young rats	n/a		100	Lys + SAA	[39]
			Adult rats	n/a		98	Lys + SAA	[39]
		Alkaline/heat-treated (75 °C, 180 min)	Young rats	n/a		48	SAA	[39]

Product	Additional information	Post-processing of product	Type of study	Crude protein (% wet matter)	DIAAS	PDCAAS	Limiting AA	References
		Alkaline/heat-treated (75 °C, 180 min)	Adult rats	n/a		35	SAA	[39]
			Weanling rats	n/a		101	Lys, SAA	[38]
		Alkaline/heat-treated (75 °C, 180 min)	Weanling rats	n/a		49	SAA	[38]
**Tofu**			Adult minipigs	10.9	75		SAA	[45]
			Rats	n/a	52	56	n/a	[46]
			In-vitro	11.3		103	Lys	[47]
		Fried (170 °C, 20 sec)	*In-vitro*	21.2		78	Lys	[47]
**Soymilk**		Ultra-high processed (UHT)	Adult minipigs	3.8	91		Lys	[45]

Supplementary Table 5: Digestible indispensable amino acid scores (DIAAS) (mean ± SD) for total soy and non-post-processed[1] soybean meal obtained from studies in growing and weanling pigs.

**Study conditions (number of datasets)**	**DIAAS**
Total soy, growing pigs (n=50)	85.6 ± 12.3
Total soy, weanling pigs (n=26)	81.9 ± 7.5
Total non-post-processed soy, growing pigs (n=37)	87.4 ± 10.7
Tot non-post-processed soy, weanling pigs (n=14)	85.2 ± 5.3
Non-post-processed soybean meal, growing pigs (n=27)	88.5 ± 10.6
Non-post-processed soybean meal, weanling pigs (n=7)	87.1 ± 3.1

[1]*Assumed since no post-processing treatments were mentioned in the original articles.*

Supplementary Table 6: Protein digestibility corrected amino acid scores (PDCAAS) (mean ± SD) for total soy and non-post-processed[1] soy protein isolate obtained from studies in adult and weanling rats.

**Study conditions** (number of datasets)	**PDCAAS**
Total soy, adult rats (n=11)	73.7 ± 21.3
Total soy, weanling rats (n=21)	90.8 ± 16.2
Total non-post-processed soy, adult rats) (n=6)	85.7 ± 17.6
Total non-post-processed soy, weanling rats (n=13)	95.8 ± 8.6
Non-post-processed soy protein isolate, adult rats (n=4)	96.0 ± 6.2
Non-post-processed soy protein isolate, weanling rats (n=9)	97.1 ± 7.0

[1]*Assumed since no post-processing treatments were mentioned in the original articles*.

References Supplementary Material

1. FAO. *Dietary protein quality evaluation in human nutrition. Report of an FAQ Expert Consultation.* FAO Food Nutr Pap, 2013. **92**: p. 1-66.
2. FAO/WHO/UNU Expert Consulation. *Energy and protein requirements Report of a Joint FAO/WHO/UNU Expert Consultation*, in *WHO Technical Report Series No. 724.* 1985, WHO: Geneva.
3. Stone AK, Nosworthy MG, Chiremba C, House JD, Nickerson MT. *A comparative study of the functionality and protein quality of a variety of legume and cereal flours.* Cereal Chemistry, 2019. **96**(6): p. 1159-1169. doi: 10.1002/cche.10226
4. Aderibigbe AS, Park CS, Adebiyi A, Olukosi OA, Adeola O. *Digestibility of Amino Acids in Protein-Rich Feed Ingredients Originating from Animals, Peanut Flour, and Full-Fat Soybeans Fed to Pigs.* Animals, 2020. **10**(11): p. 2062. doi: 10.3390/ani10112062
5. NRC, *Nutrient Requirements of Swine: Eleventh Revised Edition.* 2012, The National Academies Press: Washington, DC. https://doi.org/10.17226/13298
6. CVB, *Chemical compositions and nutritional values of feed ingredients*. 2016, Centraal Veevoeder Bureau: Wageningen, the Netherlands
7. Kaewtapee C, Eklund M, Wiltafsky M, Piepho H-P, Mosenthin R, Rosenfelder P. *Influence of wet heating and autoclaving on chemical composition and standardized ileal crude protein and amino acid digestibility in full-fat soybeans for pigs1,2.* Journal of Animal Science, 2017. **95**(2): p. 779-788. doi: 10.2527/jas.2016.0932
8. AFZ, Ajinomoto Eurolysine, Aventis Animal Nutrition, INRA, ITCF, 2000. AmiPig, *Ileal standardised digestibility of amino acids in feedstuffs for pigs.*
9. Agengo FB, Serrem CA,Wamunga FW. *Evaluation of protein nutritional quality of four soybean varieties grown in Western Kenya.* Journal of Food Research, 2018. **7**(5): p. 69-78. doi: 10.5539/jfr.v7n5p69
10. Cervantes-Pahm SK, Stein HH. *Effect of dietary soybean oil and soybean protein concentration on the concentration of digestible amino acids in soybean products fed to growing pigs1.* Journal of Animal Science, 2008. **86**(8): p. 1841-1849. doi: 10.2527/jas.2007-0721
11. Urbaityte R, Mosenthin R, Eklund M, Piepho H-P, Sauer N, Rademacher M. *Standardised ileal crude protein and amino acid digestibilities in protein supplements for piglets.* Archives of Animal Nutrition, 2009. **63**(5): p. 356-378. doi: 10.1080/17450390903052631
12. Sauvant D, Perez JM, Tran G. *Tables of composition and nutritional value of feed materials: pigs, poultry, cattle, sheep, goats, rabbits, horses and fish.* 2004, Wageningen Academic Publishers.
13. Pires CV, Oliveira MGDA, Rosa JC, Costa NMB. *Nutritional quality and chemical score of amino acids from different protein sources. .* Food Science and Technology, 2006. **26**(1): p. 179-187. doi: 10.1590/S0101-20612006000100029
14. Rodriguez DA, Lee SA, De Godoy MRC, Stein HH. *Extrusion of soybean hulls does not increase digestibility of amino acids or concentrations of digestible and metabolizable energy when fed to growing pigs.* Translational Animal Science, 2020. **4**(3). doi: 10.1093/tas/txaa169

15. Woyengo TA, Jha R, Beltranena E, Pharazyn A, Zijlstra RT. *Nutrient digestibility of lentil and regular- and low-oligosaccharide, micronized full-fat soybean fed to grower pigs1.* Journal of Animal Science, 2014. **92**(1): p. 229-237. doi: 10.2527/jas.2013-6555
16. Berrocoso JD, Rojas OJ, Liu Y, Shoulders J, González-Vega JC, Stein HH. *Energy concentration and amino acid digestibility in high-protein canola meal, conventional canola meal, and soybean meal fed to growing pigs.* Journal of Animal Science, 2015. **93**(5): p. 2208-2217. doi: 10.2527/jas.2014-8528
17. Le Thanh BV, Beltranena E, Zhou X, Wang LF, Zijlstra RT. *Amino acid and energy digestibility of Brassica napus canola meal from different crushing plants fed to ileal-cannulated grower pigs.* Animal Feed Science and Technology, 2019. **252**: p. 83-91. doi: 10.1016/j.anifeedsci.2019.04.008
18. Baker KM, Stein HH. *Amino acid digestibility and concentration of digestible and metabolizable energy in soybean meal produced from conventional, high-protein, or low-oligosaccharide varieties of soybeans and fed to growing pigs1.* Journal of Animal Science, 2009. **87**(7): p. 2282-2290. doi: 10.2527/jas.2008-1414
19. Navarro DMDL, Liu Y, Bruun TS, Stein HH. *Amino acid digestibility by weanling pigs of processed ingredients originating from soybeans, 00-rapeseeds, or a fermented mixture of plant ingredients.* Journal of Animal Science, 2017. **95**(6): p. 2658-2669. doi: 10.2527/jas.2016.1356
20. González-Vega JC, Stein HH. *Amino acid digestibility in canola, cottonseed, and sunflower products fed to finishing pigs.* Journal of Animal Science, 2012. **90**(12): p. 4391-4400. doi: 10.2527/jas.2011-4631
21. Son AR, Park CS, Park KR, Kim BG. *Amino acid digestibility in plant protein sources fed to growing pigs.* Asian-Australasian journal of animal sciences, 2019. **32**(11): p. 1745-1752. doi: 10.5713/ajas.19.0037
22. Wang JP, Kim JD, Kim JE, Kim IH. *Amino acid digestibility of single cell protein from Corynebacterium ammoniagenes in growing pigs.* Animal Feed Science and Technology, 2013. **180**(1): p. 111-114. doi: 10.1016/j.anifeedsci.2012.12.006
23. Hulshof TG, Bikker P, Van Der Poel AFB, Hendriks WH. *Assessment of protein quality of soybean meal and 00-rapeseed meal toasted in the presence of lignosulfonate by amino acid digestibility in growing pigs and Maillard reaction products.* Journal of Animal Science, 2016. **94**(3): p. 1020-1030. doi: 10.2527/jas.2015-9700
24. Petersen GI, Liu Y, Stein HH. *Coefficient of standardized ileal digestibility of amino acids in corn, soybean meal, corn gluten meal, high-protein distillers dried grains, and field peas fed to weanling pigs.* Animal Feed Science and Technology, 2014. **188**: p. 145-149. doi: 10.1016/j.anifeedsci.2013.11.002
25. Kim K, Goel A, Lee S, Choi Y, Chae B-J. *Comparative ileal amino acid digestibility and growth performance in growing pigs fed different level of canola meal.* Journal of Animal Science and Technology, 2015. **57**(1): p. 21. doi: 10.1186/s40781-015-0055-3
26. Rojas OJ, Stein HH. *Concentration of digestible, metabolizable, and net energy and digestibility of energy and nutrients in fermented soybean meal, conventional soybean meal, and fish meal fed to weanling pigs.* Journal of Animal Science, 2013. **91**(9): p. 4397-4405. doi: 10.2527/jas.2013-6409

27. Oliveira MS, Stein HH. *Digestibility of energy, amino acids, and phosphorus in a novel source of soy protein concentrate and in soybean meal fed to growing pigs.* Journal of Animal Science, 2016. **94**(8): p. 3343-3352. doi: 10.2527/jas.2016-0505
28. Liu Y, Song M, Maison T, Stein HH. *Effects of protein concentration and heat treatment on concentration of digestible and metabolizable energy and on amino acid digestibility in four sources of canola meal fed to growing pigs.* Journal of Animal Science, 2014. **92**(10): p. 4466-4477. doi: 10.2527/jas.2013-7433
29. Liu Y, Jaworski NW, Rojas OJ, Stein HH. *Energy concentration and amino acid digestibility in high protein canola meal, conventional canola meal, and in soybean meal fed to growing pigs.* Animal Feed Science and Technology, 2016. **212**: p. 52-62. doi: 10.1016/j.anifeedsci.2015.11.017
30. Jondreville C, Broecke JVD, Gâtel F, Grosjean F, Cauwenberghe SV, Sève B. *Ileal amino acid digestibility and estimates of endogenous amino acid losses in pigs fed rapeseed meal, sunflower meal and soybean meal.* Canadian Journal of Animal Science, 2000. **80**(3): p. 495-506. doi: 10.4141/a99-104
31. Cervantes-Pahm SK, Stein HH. *Ileal digestibility of amino acids in conventional, fermented, and enzyme-treated soybean meal and in soy protein isolate, fish meal, and casein fed to weanling pigs1.* Journal of Animal Science, 2010. **88**(8): p. 2674-2683. doi: 10.2527/jas.2009-2677
32. Kong C, Kang HG, Kim BG, Kim KH. *Ileal digestibility of amino acids in meat meal and soybean meal fed to growing pigs.* Asian-Australasian journal of animal sciences, 2014. **27**(7): p. 990-995. doi: 10.5713/ajas.2014.14217
33. Lee SA, Ahn JY, Son AR, Kim BG. *Standardized ileal digestibility of amino acids in cereal grains and co-products in growing pigs.* Asian-Australasian journal of animal sciences, 2020. **33**(7): p. 1148-1155. doi: 10.5713/ajas.19.0449
34. Kaewtapee C, Mosenthin R, Nenning S, Wiltafsky M, Schäffler M, Eklund M, et al. *Standardized ileal digestibility of amino acids in European soya bean and rapeseed products fed to growing pigs.* J Anim Physiol Anim Nutr (Berl), 2018. **102**(2): p. e695-e705. doi: 10.1111/jpn.12814
35. Stein HH, Kim SW, Nielsen TT, Easter RA. *Standardized ileal protein and amino acid digestibility by growing pigs and sows.* Journal of Animal Science, 2001. **79**(8): p. 2113-2122. doi: 10.2527/2001.7982113x
36. Zhang HY, Yi JQ, Piao XS, Li PF, Zeng ZK, Wang D, et al. *The Metabolizable Energy Value, Standardized Ileal Digestibility of Amino Acids in Soybean Meal, Soy Protein Concentrate and Fermented Soybean Meal, and the Application of These Products in Early-weaned Piglets.* Asian-Australasian journal of animal sciences, 2013. **26**(5): p. 691-699. doi: 10.5713/ajas.2012.12429
37. Graham A, Derouchey J, Goodband R, Tokach M, Dritz S, Thaler R. *Amino acid digestibility and energy concentration of fermented soybean meal and camelina meal for swine.* Kansas Agricultural Experiment Station Research Reports, 2013: p. 246-255. doi: 10.4148/2378-5977.7035
38. Sarwar G. *The protein digestibility-corrected amino acid score method overestimates quality of proteins containing antinutritional factors and of poorly digestible proteins*

supplemented with limiting amino acids in rats. J Nutr, 1997. **127**(5): p. 758-64. doi: 10.1093/jn/127.5.758

39. Gilani GS, Sepehr E. *Protein Digestibility and Quality in Products Containing Antinutritional Factors Are Adversely Affected by Old Age in Rats.* The Journal of Nutrition, 2003. **133**(1): p. 220-225. doi: 10.1093/jn/133.1.220
40. Mathai JK, Liu Y, Stein HH. *Values for digestible indispensable amino acid scores (DIAAS) for some dairy and plant proteins may better describe protein quality than values calculated using the concept for protein digestibility-corrected amino acid scores (PDCAAS).* British Journal of Nutrition, 2017. **117**(4): p. 490-499. doi: 10.1017/S0007114517000125
41. Hughes GJ, Ryan DJ, Mukherjea R, Schasteen CS. *Protein Digestibility-Corrected Amino Acid Scores (PDCAAS) for Soy Protein Isolates and Concentrate: Criteria for Evaluation.* Journal of Agricultural and Food Chemistry, 2011. **59**(23): p. 12707-12712. doi: 10.1021/jf203220v
42. Rutherfurd SM, Fanning AC, Miller BJ, Moughan PJ. *Protein Digestibility-Corrected Amino Acid Scores and Digestible Indispensable Amino Acid Scores Differentially Describe Protein Quality in Growing Male Rats.* The Journal of Nutrition, 2014. **145**(2): p. 372-379. doi: 10.3945/jn.114.195438
43. Sun M, Mu T, Zhang M, Arogundade LA. *Nutritional assessment and effects of heat processing on digestibility of Chinese sweet potato protein.* Journal of Food Composition and Analysis, 2012. **26**(1): p. 104-110. doi: 10.1016/j.jfca.2012.03.008
44. Fleddermann M, Fechner A, Rößler A, Bähr M, Pastor A, Liebert F, et al. *Nutritional evaluation of rapeseed protein compared to soy protein for quality, plasma amino acids, and nitrogen balance – A randomized cross-over intervention study in humans.* Clinical Nutrition, 2013. **32**(4): p. 519-526. doi: 10.1016/j.clnu.2012.11.005
45. Reynaud Y, Buffière C, Cohade B, Vauris M, Liebermann K, Hafnaoui N, et al. *True ileal amino acid digestibility and digestible indispensable amino acid scores (DIAASs) of plant-based protein foods.* Food Chemistry, 2021. **338**: p. 128020. doi: 10.1016/j.foodchem.2020.128020
46. Marinangeli CPF, House JD. *Potential impact of the digestible indispensable amino acid score as a measure of protein quality on dietary regulations and health.* Nutr Rev, 2017. **75**(8): p. 658-667. doi: 10.1093/nutrit/nux025
47. Anwar D, El-Chaghaby G. *Nutritional quality, amino acid profiles, protein digestibility corrected amino acid scores and antioxidant properties of fried tofu and seitan.* 2019, 2019. **18**(3).
